# NSP2 gene variation of the North American genotype of the Thai PRRSV in central Thailand

**DOI:** 10.1186/1743-422X-7-340

**Published:** 2010-11-24

**Authors:** Roongtham Kedkovid, Suparlark Nuntawan Na Ayudhya, Alongkorn Amonsin, Roongroje Thanawongnuwech

**Affiliations:** 1 Department of Veterinary Pathology, Faculty of Veterinary Science, Chulalongkorn University, Bangkok 10330, Thailand; 2 Department of Veterinary Public Health, Faculty of Veterinary Science, Chulalongkorn University, Bangkok 10330, Thailand

## Abstract

Porcine reproductive and respiratory syndrome virus (PRRSV) is a major swine pathogen causing economic losses in the swine industry almost worldwide. PRRSV has been divided into 2 genotypes, the European (Type 1) and North American (Type 2) genotype, respectively and displays a large degree of genetic variability, particularly at the nonstructural protein (nsp) 2 gene. This is the first study determining genetic variation of the nsp2 of Thai PRRSV isolates. The results showed that 9 out of 10 Thai PRRSV isolates were nsp2-truncated viruses that might have evolved from a virus previously introduced in the past, but not from one recently introduced.

## Findings

Porcine reproductive and respiratory syndrome virus (PRRSV) is a major swine pathogen causing major economic losses in the swine industry worldwide. PRRSV is an enveloped virus with an ssRNA genome of positive polarity belonging to the order *Nidovirales*, family *Arteriviridae*, genus *Arterivirus*. PRRSV has been genetically divided into 2 genotypes, the European (Type 1) and North American (Type 2) genotype, respectively. Both genotypes are highly diverse, sharing only approximately 60% nucleotide identity [1,2]. However, genetic variations within each genotype are also highly observed [3,4]. The PRRSV genome is approximately 15 kb in length and comprises 9 open reading frames (ORFs), ORF1a, ORF1b, ORF2a, ORF2b, ORF3, ORF4, ORF5, ORF6 and ORF7. ORF1a and ORF1b (~12 kb) encode 12 non-structural proteins (nsp), nsp1 - nsp12, which play major roles in viral replication [5-7]. The remaining ORFs encode structural proteins [8,9].

The nsp2-coding region is genetically the most variable area and crucial for viral replication due to its protease activity [10]. For nsp generation, as shown with the equine arteritis virus (EAV, a prototype virus of the genus *Arterivirus*) model, ORF1 is primarily translated into ORF1a and ORF1b polyprotein, and these 2 proteins are then cleaved into nsp1 - 8 and nsp1 - 12, respectively [11].

Recently, Type 2 PRRSV with a nucleotide deletion in the nsp2 coding region has been identified in USA, China, Japan, Denmark and Vietnam [4,12-15]. Following the outbreaks of swine high fever (SHF) syndrome in China, many genetic variants of the virus have been isolated. A novel nucleotide deletion in nsp2 found in those Chinese isolates was initially linked to the virulence of the virus [14]. The objective of this study was to investigate the deletion patterns of Type 2 PRRSV found in Thailand. Nine recent Thai isolates of Type 2 PRRSV (2007-2008), 07NP2, 07NP4, 78/51, 8NP46, 8NP154, 08RB1, 8NP147, 8NP148 and 8NP59 and one previous Thai isolate (01CS1/2) obtained in 2001 (Table 1) (kindly provided by the Chulalongkorn University-Veterinary Diagnostic Laboratory, CU-VDL) were included in this study. All samples were obtained from PRRSV-affected farms, located in the central region, an area of Thailand with a large pig population. According to the farm history, Type 2 PRRSV infection was endemic and clinically stable in those selected farms. Samples were collected when the appearance of respiratory symptoms in suckling and/or weaning pigs and reproductive failures were highly increased compared to the baseline.

**Table 1:** Sources of the Thai PRRSV and the deduced amino acid identity

Sample	**Location**^**a**^**/Year of collection**	GenBank Acc. No.	**Deduced amino acid identity when compared with**^**b**^		
			
			SY0608	MN184	Jnt1
07NP2	NP/2007	HM134182	72.2	63.3	67.7
07NP4	NP/2007	HM134183	72.2	63.6	67.7
08RB1	RB/2008	HM134184	69.6	60.0	64.9
01CS1/2	CS/2001	HM134188	75.6	62.9	68.6
8NP154	RB/2008	HM134185	72.1	63.9	64.9
78/51	NP/2008	HM134186	71.3	65.0	67.3
8NP59	NP/2008	HM134187	73.8	62.7	64.7
8NP46	RB/2008	HM134191	74.0	62.3	67.5
8NP147	NP/2008	HM134190	75.1	62.7	67.3
8NP148	NP/2008	HM134189	72.1	60.7	64.6

^a ^Abbreviations; NP = Nakornpathom, RB = Rachaburi, CS = Chacheungsao

^b ^Computed based on alignment of aa 296 - 735 region.

In this study, at first, 4 complete nsp2 nucleotide sequences of Thai PRRSV (07NP2, 07NP4, 08RB1 and 8NP154) from the acute re-emerging PRRSV-affected farms in central Thailand were characterized by multiple alignment with Type 2 PRRSV from other countries reported in GenBank. Based on nsp2 of VR2332, the prototype of Type 2 PRRSV, nucleotide deletion was found in all of those four Thai-PRRSV nsp2 sequences. Then, the remaining 6 nsp2 sequences of other Thai PRRSVs (78/51, 8NP59, 01CS1/2, 8NP46, 8NP147 and 8NP148) were further genetically characterized in the region covering all the nucleotide deletions found in 07NP2, 07NP4, 08RB1 and 8NP154 (nt 885 - 2,205 or aa 296 - 735). This specific area also contains most nucleotide deletion positions previously reported [4,13,15]. Nucleotide deletion was also found in 5 of those 6 partial nsp2 sequences.

Therefore, 9 out of 10 Thai PRRSVs in this study had a nucleotide deletion in the nsp2-coding region (or at least in the studied region). The size of the partial fragment (nt 885 - 2,205) of the nsp2-coding region of all Thai PRRSVs in this study was shown to be 3 - 384 nt smaller than the VR2332, except for 8NP147 which was devoid of either nucleotide deletion or insertion (Table 1).

**Figure 1:**
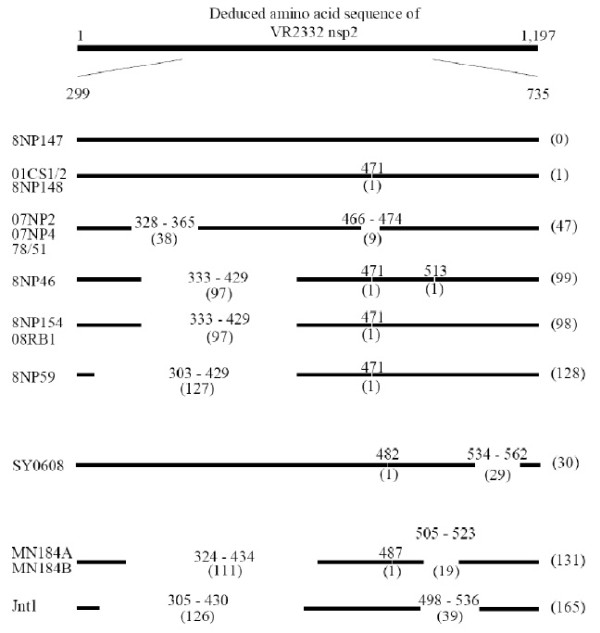
**A diagram demonstrating positions and sizes of amino acid deletions in nsp2 deduced amino acid sequences**. Horizontal lines represent deduced amino acid sequences. Names of each sequence are to the left of the horizontal lines. Gaps on the lines indicate amino acid deletions. Numbers above each horizontal line indicate positions of expected amino acid deletions. Numbers in parenthesis under each horizontal line indicate size of deletions. Numbers in parenthesis next to the horizontal lines indicate total size of amino acid deletions in the aa 299 - aa 375 region.

Based on multiple-alignment analysis of nsp2 nucleotide sequences, the results revealed possible deletion regions (Figure 1). 01CS1/2 and 8NP148 had a 3 nt (1 aa) deletion at the nt 1,411 - 1,413 position (aa 471). 07NP2, 07NP4 and 78/51 had a discontinuous 141 nt (47 aa) deletion, a 114 nt (38 aa) deletion at the nt 982 - 1,095 (aa 328 - 365) position and a 27 nt (9 aa) deletion at the nt 1,396 - 1,422 (aa 466 - 474) position. 8NP154 and 08RB1 had a discontinuous 294 nt (98 aa) deletion, a 291 nt deletion at the nt 997 - 1,287 (aa 333 - 429) position and a 3 nt (1 aa) deletion at the nt 1,411 - 1,413 (aa 471) position. 8NP46 displayed a deletion pattern resembling 8NP154 and 08RB1 but it also contained an extra 3 nt (1 aa) deletion at the nt 1,537 - 1,539 (aa 513) position. 8NP59 had a discontinuous 384 nt (128 aa) deletion, a 381 nt (127 aa) deletion at the nt 907 - 1,287 (aa 303 - 429) position and a 3 nt (1 aa) deletion at the nt 1,411 - 1,413 (aa 471) position.

**Figure 2:**
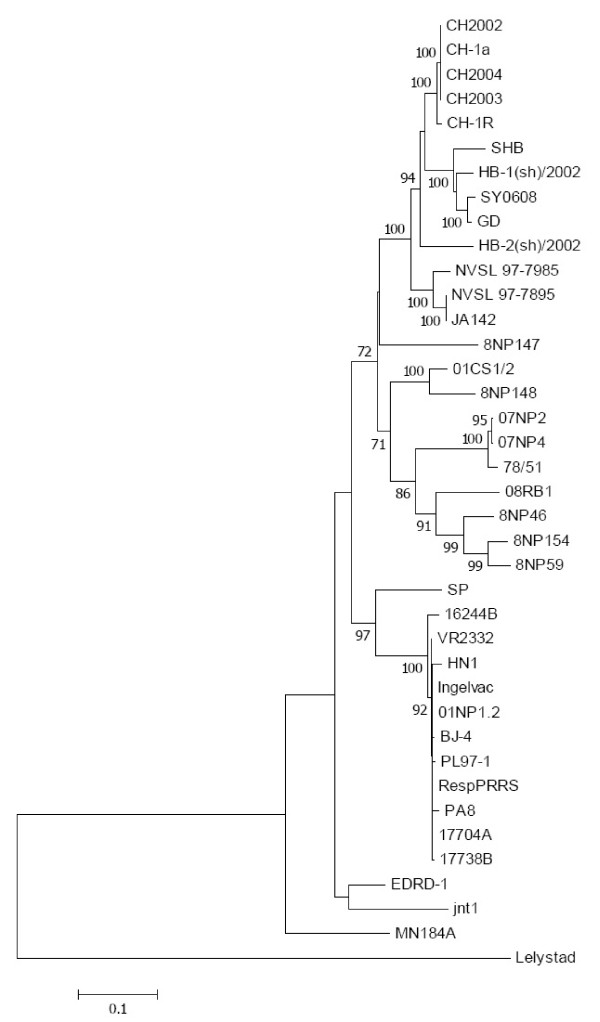
**Phylogenetic tree of nsp2 nucleotide sequences**. The tree was constructed using the nsp2 nt 886 - 2,205 region. Neighbor-joining was applied as the tree building method. Bootstrap values based on 1,000 replications are presented as numbers.

Interestingly, based on Figures 1 and 2, Thai Type 2 PRRSVs having similar deletion patterns were also located in the same cluster. Three groups of viruses were similarly identified based on both deletion patterns and phylogenetic analysis (a group of 07NP2, 07NP4 and 78/51, a group of 01CS1/2 and 8NP148, and a group of 8NP154, 08RB1, 8NP46 and 8NP59). It should be noted that 8NP147 was the only virus showing no nucleotide deletion in this study. In addition, it was located on a separate branch of the other Thai Type 2 PRRSVs. These results suggest a different evolutionary history of each PRRSV group in Thailand.

Among the studied Thai PRRSVs, sequence identities ranged from 77.0 - 99.7% and 68.1 - 99.5% for nucleotide and amino acid sequence, respectively. 07NP2 and 07NP4 showed the highest sequence identity (99.5% aa identity and 99.7% nt identity) since those two viruses had been isolated from the same farm 3 months apart showing that PRRSV still persisted and caused problems on that affected farm. The lowest sequence identity was found with 8NP147 and 8NP154 (68.1% aa identity and 77.0% nt identity). It should be noted that those isolates were from the same province.

Genetic comparison of nsp2 between Thai Type 2 PRRSVs and previously reported nsp2-truncated Type 2 PRRSVs was conducted. 8NP59, 08RB1, 8NP154 and 8NP46 displayed deletion patterns resembling other virulent isolates such as MN184A, MN184B (USA) and Jnt1 (Japan). However, sequence identity and phylogenetic studies showed no (or minor) genetic relationship. Identity of the nsp2 amino acid sequences between the Thai PRRSVs and the virulent US isolates, MN184A and MN184B ranged from 60.0 - 65.0%. Similarly, they showed only 64.6 - 68.6% identity when compared with the Japanese isolate, Jnt1 (Table 1).

**Figure 3:**
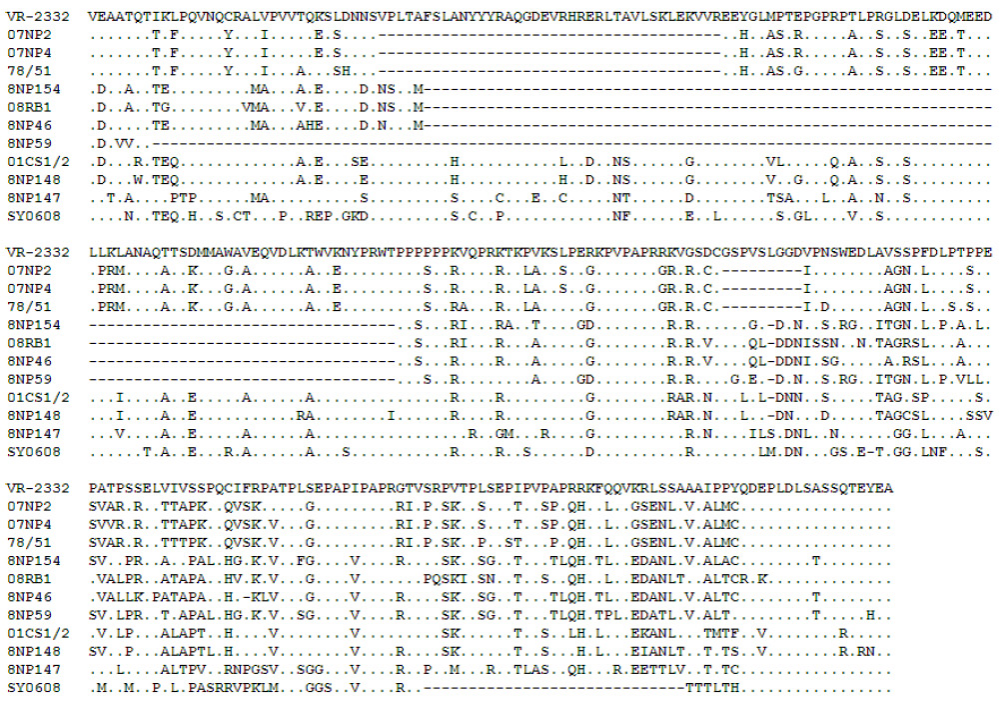
**Multiple alignment of partial nsp2 amino acid sequences**. The amino acid sequence alignment was performed using the nsp2 aa 299 - 587 region of ten Thai NA-PRRSV, SY0608 (Chinese SHF-related isolate) and VR2332. The VR2332 sequence was used as a reference. Identical amino acids and gapped positions (compared with the reference sequence) are shown as dots and hyphens, respectively.

Amino acid sequence identity between the Thai PRRSVs and SY0608 (Chinese SHF-related isolate) was low, ranging from 69.6 - 75.6% (Table 1). Sequence alignment (Figure 3) and phylogenetic tree (Figure 2) also showed no (or minor) genetic relationship among the viruses. These findings confirmed a total lack of evidence of SHF-like virus in Thailand at least at the time of sample collection. Only severe respiratory symptoms with moderate to high mortality after weaning were observed on these studied farms.

The results suggest that nsp2-truncated viruses found in this study and other nsp2-truncated viruses from other countries are unlikely to have derived from a common origin. It is more likely that the nsp2 deletion of the Thai PRRSVs has occurred in the course of individual self evolution of the PRRSVs previously circulating in Thailand.

One of the most striking characteristics of PRRSV is its genetic variation [16-19]. Nsp2 is one of PRRSV genomic regions with very high genetic variability [4,13,15,20,21]. Although the deletion in the nsp2-coding region was not related to the virulence of the emerging PRRSV in China, it could be used as a genetic marker of the highly virulent PRRSV found in China [22]. In 2007, it has been shown that the 30-aa-deletion PRRSV was also identified in Vietnam [12] which could be the result of horizontal transmission between the 2 countries. Since Thailand is in the same area as Vietnam, we therefore searched for evidence of the atypical PRRSV found in China from the acute 2007 - 2008 re-emerging PRRSV outbreaks in central Thailand. The data suggested that the atypical PRRSV having emerged in China in 2006 had not yet been introduced into Thailand, or at least into central Thailand since neither Type 2 PRRSV with the 30-aa-deletion pattern nor nucleotide sequences related to the Chinese isolates were found in this study.

At present, only 1 complete genomic sequence of the Thai Type 2 PRRSV has been reported [23]. Since the first report of PRRSV isolation in 1996, Thailand has implemented a very rigid policy aimed at imported pigs and semen having to be PRRSV-free. Thus, introduction of new exotic PRRSV strains from other countries has been limited to a minimum. Our data did not support the hypothesis of the introduction of new PRRSV strains with the same nsp2 deletion patterns from other countries. The deletion patterns found in this study could stem from the evolution of the existing PRRSVs in Thailand.

## List of abbreviations

PRRSV: porcine reproductive and respiratory syndrome virus; nsp: non-structural protein; SHF: swine high fever; ORF: open reading frame; nt: nucleotide; aa: amino acid; EAV: equine arteritis virus; PCR: polymerase chain reaction

## Competing interests

The authors declare that they have no competing interests.

## Authors' contributions

RK carried out the molecular genetic studies, sequence analysis and drafted the manuscript. AA participated in sequence alignment and phylogenetic study. SN participated in phylogenetic analysis and helped to draft the manuscript. RT conceived the study, participated in its design and helped to draft the manuscript. All authors read and approved the final manuscript.
